# Utilizing dehydroepiandrosterone sulfate and its ratio for detecting mild autonomous cortisol excess in patients with adrenal incidentaloma

**DOI:** 10.25122/jml-2023-0092

**Published:** 2023-10

**Authors:** Dheyaa Al-Waeli, Haider Alidrisi, Abbas Mansour

**Affiliations:** 1 Department of Medicine, College of Medicine, University of Thi-Qar, Nasiriyah, Thi-Qar, Iraq; 2 Thi-Qar Specialized Diabetes, Endocrine and Metabolism Center (TDEMC), Thi-Qar Health Directorate, Nasiriyah, Thi-Qar, Iraq; 3 Department of Medicine, College of Medicine, University of Basrah, Basrah, Iraq.; 4 Faiha Specialized Diabetes, Endocrine and Metabolism Center (FDEMC), University of Basrah, Basrah, Iraq.

**Keywords:** Adrenal incidentaloma, subclinical Cushing syndrome, DHEA-S ratio, DHEA-S, MACE, Mild autonomous cortisol excess

## Abstract

Subclinical Cushing syndrome is a condition of mild autonomous cortisol excess (MACE) that lacks typical features of Cushing syndrome but is associated with many complications. It represents a common hormonal dysfunction among patients with adrenal incidentaloma (AI), defined as unexpected masses or lesions found in the adrenal glands during radiological examinations of the chest or abdomen unrelated to adrenal gland assessment. The study evaluated the accuracy of dehydroepiandrosterone sulfate (DHEA-S) and dehydroepiandrosterone sulfate ratio (calculated by dividing the DHEA-S value by the age and sex-adjusted normal range of DHEA-S) in detecting MACE in AI patients. A cross-sectional study was conducted from April 2021 to July 2022 at the Faiha Specialized Diabetes, Endocrine, and Metabolism Centre (FDEMC) in Basrah, southern Iraq, involving 38 AI patients. Comprehensive laboratory and radiological evaluations were performed, including tests for adrenocorticotropic hormone (ACTH), renin, aldosterone, aldosterone/renin ratio (ARR), metanephrine, normetanephrine, cortisol, DHEA-S, and the 1-mg overnight dexamethasone suppression test (1-mg ONDST). Among the AI patients, 14% had MACE. Both DHEA-S ≤75 µg/dL and a DHEA-S ratio ≤1.7 exhibited a sensitivity of 80% each, with specificities of 73.3% and 76.6%, respectively, in diagnosing MACE in individuals aged ≤65 years. The negative predictive values were 95.7% and 95.8%, respectively. Low DHEA-S and DHEA-S ratio had high sensitivity and specificity in predicting MACE among AI patients aged ≤65 years, with strong negative predictive value.

## INTRODUCTION

Mild Autonomous Cortisol Excess (MACE), or subclinical Cushing syndrome, is the most common hormonal abnormality in patients with adrenal incidentalomas (AIs). It is associated with an elevated risk of osteoporosis, dyslipidemia, hypertension, obesity, and diabetes mellitus [1]. AIs can be defined as lesions or masses in the adrenal gland more than or equal to 10 mm and are incidentally detected during imaging procedures performed for the chest or abdomen for purposes other than adrenal gland assessment [1]. Advances in radiological techniques have led to increased detection of AIs, making them a frequent referral concern for endocrinology centers [2]. Most AIs are benign and non-functional, but in 20-40% of cases, there is insidious hormonal overproduction, such as cortisol, aldosterone, sex hormones, or catecholamines [1]. Unless apparent suggestive clinical features exist, AIs less than 10 mm need no further assessment [1]. Although MACE is associated with autonomous cortisol overproduction, causing hypothalamus–anterior pituitary-adrenal cortex axis (HPA-axis) suppression and impairing the responses to stressful conditions, there are no typical clinical features of Cushing syndrome [3]. Therefore, MACE should be excluded in all patients with AIs [1]. Many guidelines recommend the 1-mg Overnight Dexamethasone Suppression Test (ONDST) as a screening test for hypercortisolism, with a cutoff value of ≥1.8 µg/dL, achieving 100% sensitivity and 80% specificity, respectively [4].

The adrenocorticotropic hormone (ACTH) and dehydroepiandrosterone sulfate (DHEA-S) are used to support the diagnosis of MACE and to demonstrate the autonomous overproduction of cortisol in patients with AIs. Typically, the plasma level of ACTH is low or inhibited in MACE. However, ACTH has a short half-life and exhibits pulsatile secretion, which can lead to overlapping levels in healthy individuals [2, 5]. The DHEA-S is an adrenal androgen produced under the control of ACTH. Therefore, the suppression of ACTH in MACE results in a decrease in DHEA-S levels. DHEA-S has a relatively long half-life of about 10-16 hours and maintains a relatively constant status over 24 hours. This characteristic makes it a valuable indicator for assessing persistently suppressed HPA axis function due to MACE [6]. The main objective of this study was to evaluate the diagnostic accuracy of DHEA-S and DHEA-S ratio compared to the 1-mg ONDST for the diagnosis of MACE among patients with AIs.

## MATERIAL AND METHODS

### Study design

A cross-sectional study was conducted at Faiha Specialized Diabetes, Endocrine, and Metabolism Centre (FDEMC) in Basrah, southern Iraq, from April 2021 to July 2022.

### Inclusion and exclusion criteria

This study included all patients with adrenal incidentalomas referred to FDEMC for endocrine evaluation. Exclusion criteria included patients using medications affecting the dexamethasone metabolism or catecholamine production, individuals with major psychiatric disorders, alcohol consumption, those with clinical Cushing syndrome, a history of adrenal/pituitary surgery, and individuals with adrenal masses found during cancer staging.

### Clinical assessment

A detailed history was obtained from all patients, including information on drug intake, weight gain, hirsutism, palpitations, hypertension, fractures, and adrenal or pituitary surgery. Clinical examination included measurements of body mass index (BMI) (by Stadiometer SECA-763) and blood pressure in a seated position after resting for five minutes (measured by semi-automated oscillometric maneuver (Omron HEM -780). Hypertension was defined as systolic blood pressure (SBP) ≥140 mmHg or diastolic blood pressure (DBP) ≥90 mmHg [7]. Clinical features of overt Cushing syndrome, such as striae, moon face, central obesity, and proximal myopathy, were assessed.

### Laboratory assessment

Blood samples were collected in the early morning after fasting and analyzed using electrochemiluminescence immunoassay principles (ECLIA) by Cobas e411 (Roche Diagnostics, Germany) for serum cortisol, DHEA-S, and plasma ACTH. Serum 17-hydroxyprogesterone (17-OHP) levels were determined for patients with elevated DHEA-S, while plasma renin concentration, aldosterone, metanephrine, and normetanephrine were measured using enzyme-linked immunosorbent assay (ELISA) with the (DRG)R ELISA kit system (Germany). Fully automated chemical analyzer (Cobas c311, Roche Diagnostics, Germany) was utilized to assess serum for usual chemistry analysis. Plasma glycated hemoglobin (HbA1c) was measured using a high-performance liquid chromatography (HPLC) system (Bio-Rad D-10 analyzer), and the diagnosis of diabetes mellitus was made following the American Diabetic Association criteria [8]. The diagnosis of pheochromocytoma (PCC) was considered when normetanephrine or metanephrine levels were elevated four times above normal values [9]. Primary aldosteronism (PA) was diagnosed when the aldosterone/renin ratio (ARR) was >5.7 [10]. The ACTH stimulation test was used to diagnose congenital adrenal hyperplasia (CAH) in patients with high 17-OHP levels (200-1000 ng/dL), with a diagnosis established when 17-OHP exceeded 1000 ng/dL [11]. The 1-mg overnight dexamethasone suppression test (1-mg ONDST) was performed for all patients to rule out MACE, and abnormal tests were considered when morning cortisol levels were ≥1.8 µg/dL (50 nmol/L) [6]. The DHEA-S ratio was estimated by dividing the DHEA-S value by the minimum of the age and sex-adjusted normal range of DHEA-S [6].

### Radiological evaluation

Full radiological assessment of AIs was conducted using computed tomography (CT) scans. Adrenal adenomas were diagnosed in AIs with native Hounsfield units (HU) ≤10. A triphasic adrenal CT protocol was used for AIs with HU>10 to measure the absolute percentage washout (APW). Adrenal incidentalomas with APW ≥60% were classified as adenomas, while those with APW <60% were considered for surgery [1]. Myelolipomas and adrenal cysts were diagnosed using typical radiological characteristics [12].

### Statistical analysis

Data were analyzed using the Statistical Package for Social Sciences (SPSS) version 23.0. Patient characteristics, including age, BMI, DBP, SBP, and other continuous variables such as hormonal data (Cortisol, DHEA-S, 1-mg ONDST, ACTH, Renin, Aldosterone, ARR, Normetanephrine, and Metanephrine), as well as radiological parameters, were presented as mean ± standard deviation (SD). Patient ages were stratified into two groups: above or equal to 40 years and less than 40, while BMI was categorized as above or equal to 30 kg/m^2^ and less than 30 kg/m^2^. Receiver Operating Characteristic (ROC) curves were used to determine the optimal sensitivity and specificity cutoff values for DHEA-S and DHEA-S ratio in relation to MACE. Categorical variables like age, BMI groups, and AI diagnosis were summarized as numbers and frequencies N (%). Independent student t-tests and Chi-square tests were used to evaluate correlations between categorical and continuous variables. A p-value of <0.05 was considered statistically significant in comparative tests.

## RESULTS

Thirty-eight patients with a mean age of 47.6±18.3 years participated in this study, of whom 23 were female. The mean BMI was 27.7±7.2 kg/m^2^, and 17 patients (44.7%) were classified as obese (BMI≥30). These patients had 43 AIs, with 5 having bilateral AIs. Among these, two had congenital adrenal hyperplasia (CAH), one had bilateral ACTH-secreting pheochromocytoma (PCC), one had bilateral non-Hodgkin lymphoma (NHL), and one had bilateral non-functional adenoma (NFA).

**Figure 1:**
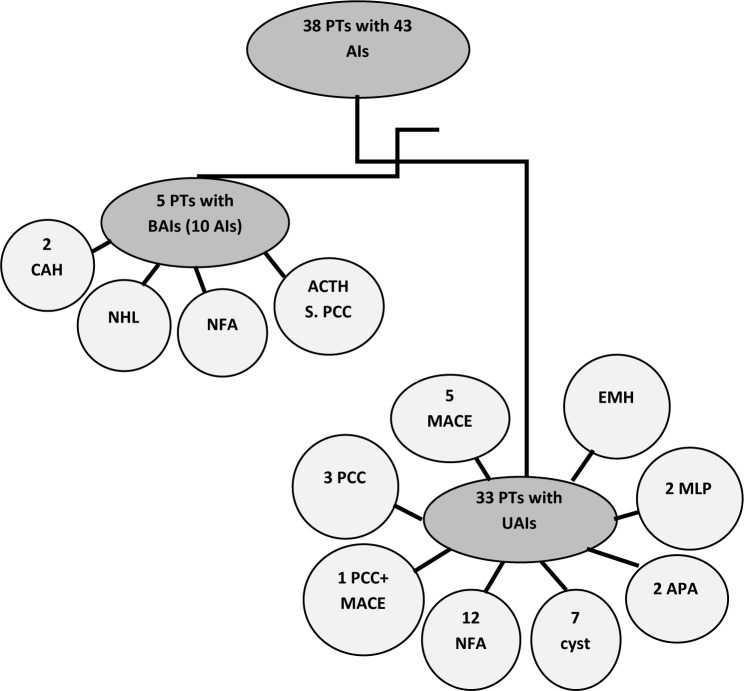
Flow chart of participants. Abbreviations: PTs, patients; AIs, Adrenal Incidentalomas; BAIs, Bilateral Adrenal Incidentalomas; CAH, Congenital Adrenal Hyperplasia; NHL, Non-Hodgkin Lymphoma; NFA, Non-Functional Adenoma; ACTH S. PCC, ACTH Secreting Pheochromocytoma; UAIs, Unilateral Adrenal Incidentalomas; MACE, Mild Autonomous Cortisol Excess; PCC, Pheochromocytoma; APA, Aldosterone Producing Adenoma; EMH, Extra-Medullary Hematopoiesis.

Regarding patients with unilateral AIs (33 patients), 21 had right-sided AIs. Six patients had MACE, with one patient having PCC co-secreting cortisol. Twelve patients had NFA, seven had adrenal cysts, four had PCC (with one co-secreting cortisol causing MACE), two had aldosterone-producing adenomas, two had myelolipomas, and one had extramedullary hematopoiesis (EMH) (Figure 1).

Patients with MACE had a significantly lower mean BMI (23.6±5.7) compared to patients with non-functioning AI (31.6±7.5) (p=0.03). Patients with MACE were also significantly younger than those with non-functioning AIs (NFAI). Approximately 50% of patients with MACE were less than 40 years old, compared to only 6.2% of patients with NFAI (p<0.01). However, there were no significant differences in gender, prevalence of hypertension, or diabetes. Moreover, 14% of patients were diagnosed with MACE using the 1-mg ONDST. Both DHEA-S and DHEA-S ratio were lower in patients with MACE compared to other AIs, but the differences were not statistically significant.

**Figure 2:**
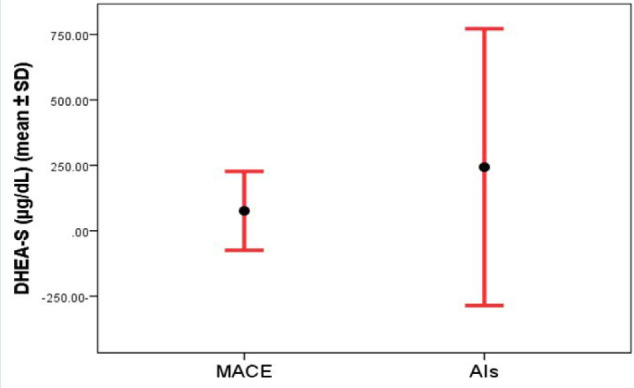
Correlation between mean DHEA-S and MACE in 65-year-old patients or younger (p=0.1)

**Figure 3:**
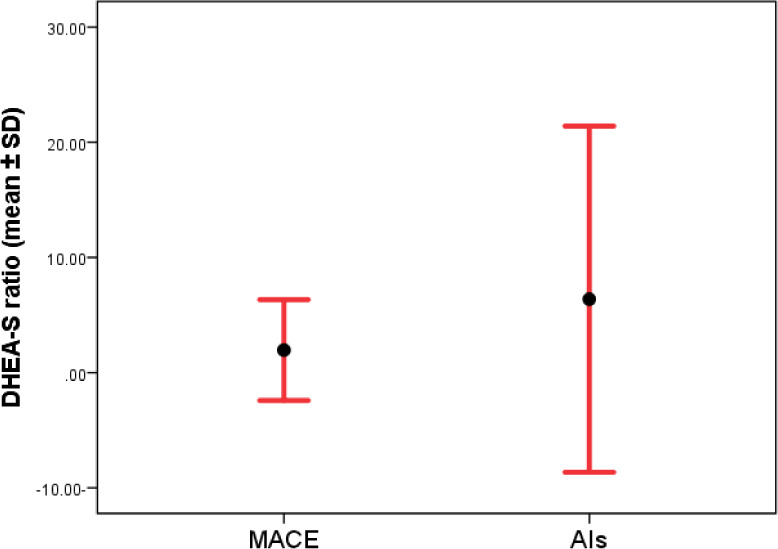
Correlation between DHEA-S ratio and MACE in 65-year-old patients or younger (p=0.01)

After excluding three elderly patients (older than 65 years) from the analysis (because the DHEA-S level reduced physiologically in this age group), a total of 35 AIs remained for comparison. These patients were 65 years or younger and had a mean age of 42.9±15.3 years, with 18 (51.4%) of them being female. Five of these 35 AIs were diagnosed with mild autonomous cortisol excess (MACE). The mean DHEA-S ratio in patients with MACE was 1.9±2.1, which was significantly lower compared to other AIs (6.2±7.4) (p=0.01). Although the mean DHEA-S (µg/dL) was lower in patients with MACE (75.9±75.3) compared to other AIs (243.1±264.4), it was not statistically significant (p=0.1) (Figures 2 and 3).

**Figure 4:**
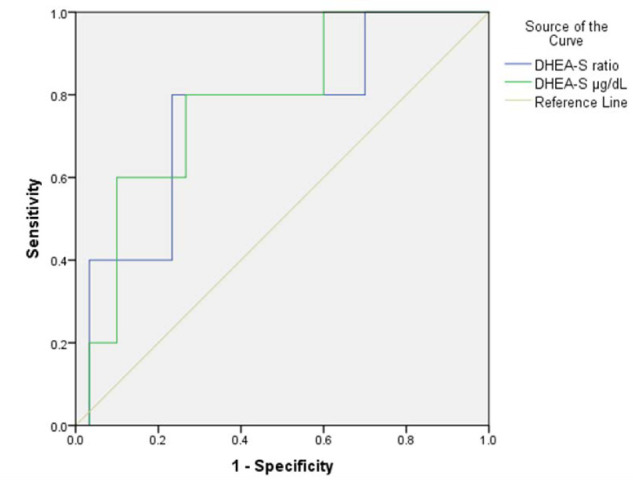
Receiver operating curve of the diagnostic accuracy of DHEA-S µg/dL levels and DHEA-S ratio for MACE patients 65 years or younger with AIs

ROC curves were plotted to assess the diagnostic accuracy of low DHEA-S and DHEA-S ratios for MACE patients with AIs. DHEA-S showed an area under the curve (AUC) of 0.78 (p=0.04, 95% CI 0.57-0.98), while DHEA-S ratio showed an AUC of 0.75 (p=0.07, 95% CI 0.51-0.98). A DHEA-S level of 75 µg/dL or less had 80% sensitivity and 73.3% specificity for MACE, and a DHEA-S ratio of 1.7 or less had 80% sensitivity and 76.6% specificity for MACE (Figure 4).

**Figure 5:**
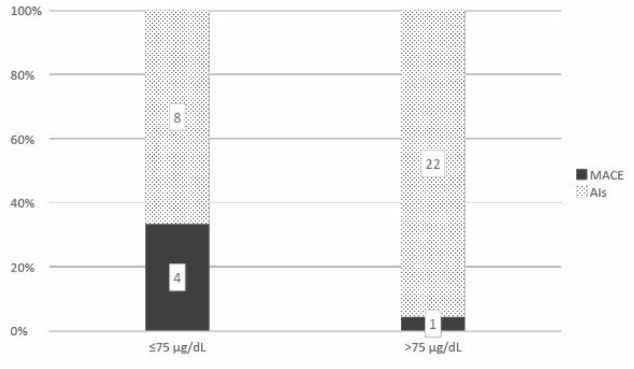
Correlation between cut-off DHEA-S of 75 µg/dL and diagnosis of MACE in patients 65 years or younger with AIs (p=0.02)

**Figure 6:**
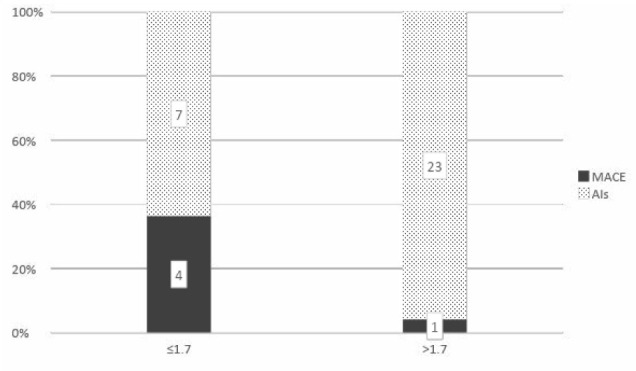
Correlation between cut-off DHEA-S ratio of (1.7) and diagnosis of MACE in patients 65 years or younger with Ais (p=0.01)

A cut-off DHEA-S level of 75 µg/dL or less and a DHEA-S ratio of 1.7 or less were significantly correlated with MACE (p=0.02 and 0.01, respectively) (Figures 5 and 6). The positive predictive values for both these cut-off values were 33.3% and 36.4%, respectively, but with high negative predictive values of 95.7% and 95.8% for MACE in patients with AIs.

Despite observing lower average DHEA-S levels and DHEA-S ratios in the MACE group compared to patients with non-functional adenomas and other adrenal incidentalomas, these differences did not reach statistical significance (Table 1). This could be due to the limited sample size, randomization, and the fact that only a single sample was taken from each patient, with no follow-up samples for further analysis.

**Table 1:** Post hoc Tukey test analysis of the DHEA-S and DHEA-S ratio among patients with MACE, NFA, and other AIs

Variables	MACE (5 patients)	NFA (13 patients)	Other AIs (17 patients)	p-value
DHEA-S(µg/dL) (mean±SD)	75.9±75.3	200.9±251.6	227.4±235.0	0.4
DHEA-S ratio (mean±SD)	1.9±2.1	3.4±2.3	5.6±7.5	0.3

Abbreviations: DHEA-S, Dehydroepiandrosterone-sulfate; MACE, Mild autonomous cortisol excess; NFA, Non-Functional Adenoma; AIs, Adrenal Incidentalomas.

## DISCUSSION

This study evaluated the sensitivity and specificity of DHEA-S and DHEA-S ratio as a simple screening test for MACE in patients with AIs, a condition known for its significant impact on cardiovascular health, bone density, and metabolic state [1]. Our findings revealed a female predominance among AI patients, with a female-to-male ratio of 1.5:1. This gender difference has been noted in numerous large-scale studies and can be attributed to increased ultrasound examinations in female patients. This gender discrepancy was not evident in autopsy studies [13].

The incidence of MACE in our study was approximately 14%, consistent with findings from other large studies [1]. We found that low DHEA-S and DHEA-S ratio are reliable screening tests for MACE with high sensitivity and specificity in those patients ≤65 years. A retrospective study conducted in the United Kingdom, which analyzed data from 185 AI patients at the Cambridge University Hospitals Foundation Trust, found that a DHEA-S ratio of 1.2 or less had a sensitivity of 99% and specificity of 91% in screening for MACE in AI patients. The results of the Cambridge study were comparable to our study but with higher sensitivity and specificity at a lower cut-off value of the DHEA-S ratio. These differences may be attributed to variations in study design and the larger sample size in the Cambridge study [6].

In the present study, both DHEA-S and DHEA-S ratio had low positive predictive values but high negative predictive values, which may indicate their greater significance in exclusion rather than confirmation of MACE. A Turkish study from Izmir retrospectively assessed 249 patients with AIs at Dokuz Eylul University and concluded that DHEA-S level of less than 40 µg/dL predicted MACE with 68% and 75% sensitivity and specificity, respectively [14].

In addition to its role in MACE screening, DHEA-S proved valuable in diagnosing congenital adrenal hyperplasia (CAH) in two cases. The diagnosis of adrenal tumors was also suggested by others in the presence of high DHEA-S levels [15].

Although ACTH was suppressed (<10 pg/mL) in half of our patients with MACE, we did not use it as a criterion for the diagnosis of MACE due to many interferences in laboratory assays [16]. The brief half-life of ACTH, diurnal variation, pulsatile behavior of secretion, and its detected levels in many patients with non-ACTH-dependent Cushing’s syndrome limit its significance in diagnosing MACE [15, 17].

A retrospective study conducted in Spain involving 197 patients with AIs, similar to our study, used the 1-mg ONDST as the gold standard for MACE diagnosis with different cut-off values but did not consider the DHEA-S ratio. This study found that DHEA-S had limited reliability as a diagnostic test for MACE, with a sensitivity of 30.3% and specificity of 79.3%, along with positive and negative predictive values of 47.9% and 64.4%, respectively [18]. In our study, we employed a lower DHEA-S cutoff value compared to the higher level used in the Spanish study, which may explain the higher sensitivity of DHEA-S, greater negative predictive value, and comparable specificity in our findings.

A prospective study in China considered a DHEA-S level of less than 60 µg/dL and a DHEA-S ratio cutoff value of 1.3 as valuable screening tests for detecting MACE, achieving good sensitivity, specificity, positive predictive value (PPV), and negative predictive value (NPV) [19]. The results of the present study were comparable with the Chinese results but at mildly higher levels for both DHEA-S and DHEA-S ratio, which could be due to differences in sample size, different population characteristics, and randomization process.

Some of the limitations of this study include the small size, short duration, and single-center design. Similar to other screening tests, DHEA-S may have false-positive results in patients with suppressed ACTH levels, such as those with chronic opioid or glucocorticoid use or hypothalamic or pituitary dysfunctions.

## CONCLUSION

In this study, a substantial portion of adrenal incidentalomas (AIs) were non-functional, with mild autonomous cortisol excess (MACE) being the most prevalent functional subtype. The results indicate that DHEA-S and the DHEA-S ratio can serve as valuable diagnostic tools for assessing patients aged 65 or younger with AIs. Elevated levels of either marker demonstrated a strong negative predictive value for excluding MACE in this patient group.

## Personal thanks

All authors are grateful to the laboratory personnel of FDEMC for their assistance and encouragement.
